# Tp-Te Interval and Tp-Te/QT Ratio Are Valuable Tools in Predicting Poor Outcome in Sepsis Patients

**DOI:** 10.3389/fcvm.2022.879085

**Published:** 2022-05-23

**Authors:** Duo Li, Yibing Weng, Genshen Zhen, Li Jiang

**Affiliations:** ^1^Department of Critical Care Medicine, Fuxing Hospital, Capital Medical University, Beijing, China; ^2^Department of Critical Care Medicine, Beijing Luhe Hospital, Capital Medical University, Beijing, China; ^3^Department of Critical Care Medicine, Xuanwu Hospital, Capital Medical University, Beijing, China

**Keywords:** sepsis, sepsis-induced myocardial dysfunction, transmural dispersion repolarization (TDR), Tp-Te interval, Tp-Te/QT

## Abstract

**Objective:**

About 50% of patients with sepsis have different degrees of myocardial inhibition, known as sepsis-induced myocardial dysfunction (SMD), which increases the mortality rate of sepsis. Tp-Te interval and Tp-Te/QT ratio reflect ventricular transmural dispersion repolarization (TDR), and have good predictive value for death in patients with heart disease. This study aimed to investigate the prognostic value of Tp-Te and Tp-Te/QT in patients with sepsis.

**Methods:**

The current study included a total of 625 participants: 201 patients with sepsis, 213 patients with heart failure, and 211 healthy participants. According to the severity and outcome, the patients with sepsis were divided into the sepsis group and the septic shock group, and the death group and the survival group to explore the differences of indicators among subgroups of sepsis. The ROC curve was used to analyze the predictive value of the indicators for deaths of patients with sepsis and calculate the cutoff point. Then, we investigated the incidence of arrhythmia in patients with sepsis with different TDR. The correlation between Tp-Te/QT and the commonly used predictive indicators in ICU was also discussed.

**Results:**

(1) Tp-Te and Tp-Te/QT in patients with sepsis and heart failure (HF) were significantly higher than the control group (*p* < 0.01). (2) Compared with patients with sepsis, the increase of Tp-Te and Tp-Te/QT is more prominent in patients with HF. Especially, the increase of the Tp-Te/QT was statistically significant (*p* < 0.001). (3) compared with patients with sepsis (no shock), the Tp-Te, Tp-Te/QT, and SOFA were increased in patients with septic shock (*p* < 0.05). (4) In the death group, Tp-Te /QT, SOFA, and Apache-II were higher; LVEF was lower than the survival group (*p* < 0.05). (5) ROC curves showed that Tp-Te/QT, SOFA, and LVEF have predictive values for death (*p* < 0.05; AUC = 0.808, 0.716, 0.412). The cutoff point of Tp-Te/QT was 0.32. (6) The incidence of arrhythmia is different in patients with sepsis with different TDR. (7) There is a significant correlation between Tp-Te/QT and SOFA (*p* < 0.001, *r* = 0.79).

**Conclusion:**

TDR in patients with sepsis is significantly increased, which was between healthy population and patients with HF. Tp-Te and Tp-Te/QT are effective indicators to reflect the severity and poor outcome of patients with sepsis.

## Background

Sepsis is life-threatening organ dysfunction caused by a dysregulated host response to infection, which affects millions of people worldwide every year (1–3). As the center of the circulatory system, the heart is the target organ easily affected by sepsis. Nearly 50% of patients with sepsis have different degrees of myocardial inhibition, known as sepsis-induced myocardial dysfunction (SMD). Previous studies have shown that SMD is likely to increase the mortality rate of patients with sepsis from 20–50% to 70–90% (4). Therefore, early identification, diagnosis, and timely therapy show a promising prognosis of sepsis.

However, the clinical diagnostic criteria for SMD are not unified. The reason is that the clinical manifestations of SMD are very complex, including systolic failure and diastolic failure, which can be either left ventricular and right ventricular failure or in combination. Therefore, the early diagnosis of SMD is challenging for clinicians, and there is no clinical “gold standard.” At present, the commonly used indicators of septic cardiac dysfunction include BNP, NT-proBNP, and echocardiographic indicators, such as LVEF, *E*/*E*′, etc.

ECG index opens a new perspective to explore SMD. Tp-Te interval and Tp-Te/QT ratio, the indicators of ventricular transmural dispersion repolarization (TDR) (5, 6), have good predictive value for death in patients with coronary atherosclerotic heart disease with chronic heart failure and Brugada syndrome. It also has an excellent predictive effect on the occurrence of malignant ventricular arrhythmias (torsade de pointes, ventricular fibrillation) (7–12). Recently, some pediatricians have found that Tp-Te/QT is an independent predictor of death in children with sepsis (13), which may be related to the increased mortality in children with SMD. However, there are few studies in the field of adult sepsis.

This is a prospective observational study aimed to investigate the prognostic value of electrocardiology indicators in adult patients with sepsis.

## Study Population

### Sepsis Group

A total of 3,136 patients were admitted to the ICU of Beijing Luhe Hospital, Capital Medical University, from January 2018 to May 2021. Approximately, 886 patients diagnosed with sepsis were enrolled in this prospective study. The diagnostic criteria met the definition of sepsis 3.0 jointly issued by the Society of Critical Care Medicine (SCCM) and the European Society of Intensive Care Medicine (ESICM) in February 2016 (14).

### Heart Failure Group

Patients with heart failure (HF) were recruited from age- and gender-matched patients hospitalized in the first ward of the cardiology department and ICU during the same period. The diagnosis meets the criteria for acute heart failure of the European Society of Cardiology (ESC) 2016 (15).

### Control Group

Healthy controls were selected from the same period in the physical examination center of Beijing Luhe Hospital, with no abnormalities in electrocardiogram and echocardiography, and matched in age and sex.

### Inclusion and Exclusion Criteria

Inclusion criteria for the sepsis and heart failure groups were: (1) expected length of stay >48 h; (2) age, ≥18 years.

Exclusion criteria for the sepsis group: Patients with a previous diagnosis of arrhythmia, cardiac insufficiency, coronary heart disease, hypertension, or cardiomyopathy. These types of heart disease may affect the QT interval.

Exclusion criteria for the heart failure group: Patients with conduction block, stress syndrome, temporary or permanent pacemaker implantation.

Exclusion criteria for all subjects: (1) Pregnant patients; (2) taking antiarrhythmic drugs affecting QT interval within 1 week before admission and before electrocardiogram; (3) patients with uncorrected electrolyte disturbances before electrocardiogram. (The normal values of electrolyte in this study were defined as follows: arterial blood potassium, 3.4–4.5 mmol/l; calcium, 1.15–1.29 mmol/l; sodium, 136–146 mmol/l; chlorine, 98–106 mmol/l; venous blood magnesium, 0.75–1.0 mmol/l.); (4) refused to participate in the study for any reason.

A total of 625 subjects were included in the study: 201 patients with sepsis, 213 patients with HF, and 211 healthy persons.

## Methods

### General Data Collection

The general information, including age, sex, medical history, and medication history, was recorded within 24 h upon admission.

BNP test and bedside cardiac examinations were completed for patients with sepsis and HF within 48 h of admission. If two or more BNP values are detected, we use the maximum value within 48 h. BNP was detected using Alere Triage MeterPro quantitative fluorescence immunoassay. The SonoSite Edge II Ultrasound diagnostic instrument was used for cardiac ultrasound examination, and the Simpson biplane method to measure left ventricular ejection fraction (LVEF).

For the sepsis group, sequential organ failure assessment (SOFA) and acute physiology and chronic health evaluation (Apache-II) were recorded at three time points: 0 h (immediately after admission), 24 h, and 48 h, and obtained the maximum score.

### Electrocardiology Indicators

All electrocardiology indexes collected in this study were obtained using ECG-1350C (Shanghai Optoelectronic Medical Electronic Instrument) with a paper speed of 25 mm/s, and amplitude of 10/mV.

#### Time and Frequency of Electrocardiography

A single ECG recorded at the physical examination center was selected for measurement in the control group. In patients with HF and sepsis, three ECG recordings were measured at 0 h, 24 h, and 48 h.

#### Selection of Measurement Leads

Current studies on the Tp-Te interval of large-sample normal electrocardiogram (16) suggest that the maximum Tp-Te interval of the 12-lead electrocardiogram is mainly distributed in the chest lead, especially in leads V2-V4, which has a strong concentration, and this distribution has good repeatability in men and women. Therefore, we measured the QT interval and Tp-Te interval of three consecutive cardiac cycles in leads V2, V3, and V4 for each study object and ECG. Then, the average value was obtained.

#### Heart Rate

The heart rate of the control group was recorded in physical examination. The heart rate of patients with HF was the mean of three heart rates recorded at 0, 24, and 48 h. Heart rate in patients with sepsis was measured using the mean hourly heart rate recorded on the ICU nursing note sheet within 48 h of admission.

#### T-peak

T-peak is the peak point of the positive T wave or the valley point of the negative T wave.

#### T-end

(1) If the intersection point between the descending branch of T wave and the equipotential line is clear, the intersection point is considered Te; (2) If the intersection is not clear, the intersection of the T wave down a tangent and the equipotential is considered Te; (3) If U wave appears after T wave, the lowest point at the junction of T wave and U wave is taken as the endpoint of T wave; If U and T waves partially merge, make the extension line of T wave descending branch, and take the intersection point of the extension line and the baseline. (4) Flat and bidirectional T waves are not included in the analysis. The baseline floatation and interference subjects were immediately repeated with electrocardiograms till measurement.

#### Tp-Te Interval

The time distance between T-peak to T-end represents the transmural dispersion repolarization (TDR) of the ventricle.

#### QT Interval

The distance between the beginning of the Q wave and the end of T wave represents the entire process of ventricular depolarization and repolarization.

#### Computing Method

(1) In healthy controls (take QT as an example): Five consecutive cardiac cycles were selected in the V2 lead to measure five QT intervals and calculate the average amount QTmean-V2. Likewise, QTmean-V3 and QTmean-V4 are calculated on leads V3 and V4. Then, the mean values of QTmean-V2, QTmean-V3, and QTmean-V4 were calculated as the “QT interval” values for index analysis.

(2) In the sepsis and HF groups (take QT as an example): Immediately upon admission, choose leads V2, V3, and V4 to measure the QT interval of five consecutive cardiac cycles, and the mean QTmean-v2, QTmean-V3, and QTmean-v4 were taken. Then, calculate the average of the three and record it as QTmean-0 h. Similarly, measure and calculate QTmean-24 h and QTmean-48 h. The mean value of the three factors was calculated as the “QT interval” value of the research object for index analysis.

(3) The Tp-Te interval was calculated using the same method described above.

#### Tp-Te/QT

The ratio of Tp-Te to QT. This ratio can eliminate the effect of heart rate on the QT and Tp-Te interval.

All the electrocardiology indicators were measured by the same experimenter who were familiar with measurement standards, combined with the average method to reduce the random error.

### Statistical Analysis

SPSS21.0 was used for statistical analysis, the confidence interval was 95%, and the test level was 0.05. We evaluated the distribution pattern of numerical data using the Kolmogorov–Smirnov test. Data are expressed as mean ± SD, median (interquartile range) or number (percentage) where appropriate. We used Student's *T*-test to compare normally distributed numerical data for independent groups. If there were more than two groups, we used one-way variance analyses, Bonferroni test. We use the Mann–Whitney U-test to compare two groups with abnormally distributed numerical data, and if there were more than two groups, we used the Kruskal–Wallis test. ROC curve was used to analyze the prognostic value of different indicators for patients with sepsis and calculate the cutoff value. Pearson's correlation test was used to evaluate the correlation between electrocardiology index and traditional indicators.

## Results

### Study Participants

**Figure 1:**
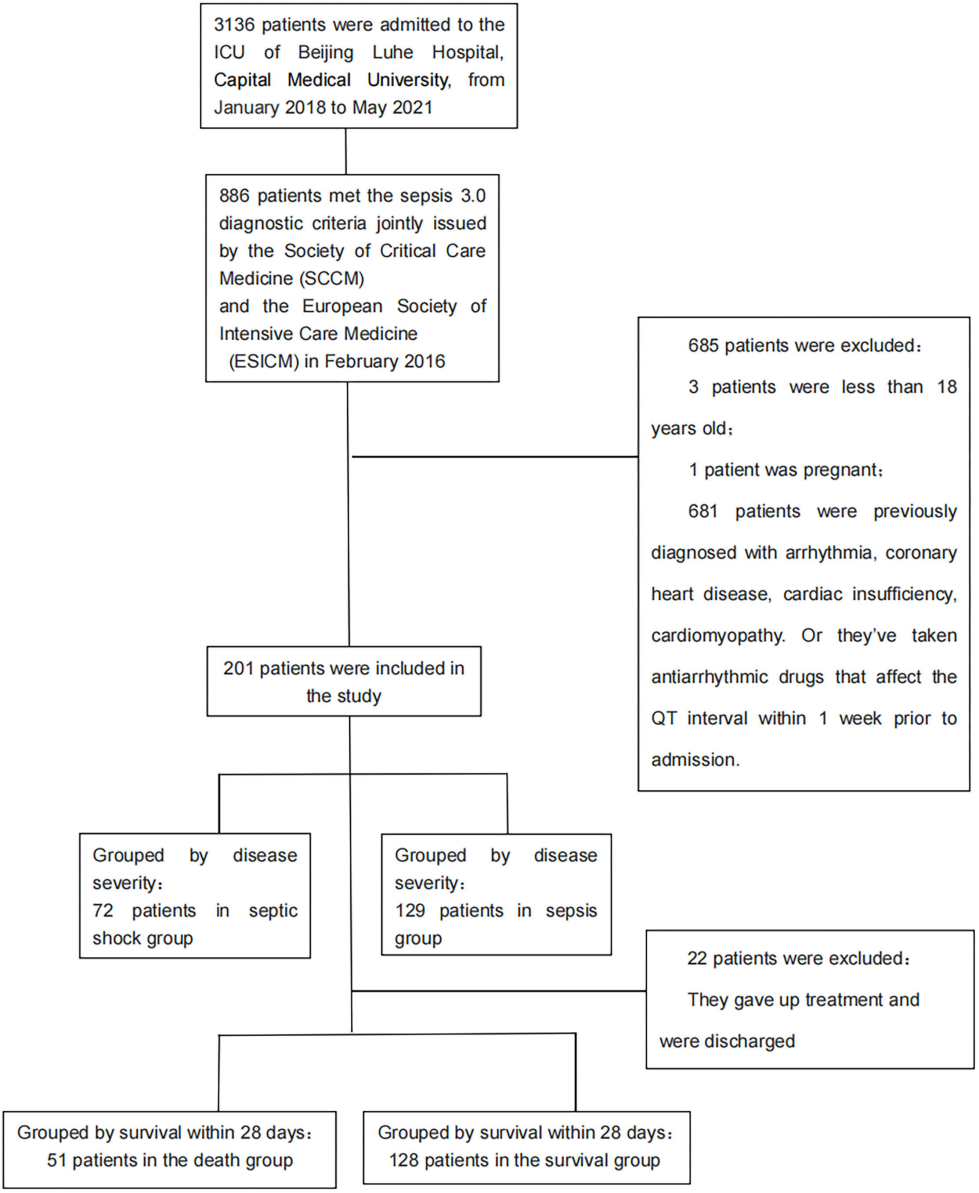
Flow diagram of sepsis patients selection.

A total of 3,136 patients were admitted to the ICU of Beijing Luhe Hospital from January 2018 to May 2021. Among them, 886 patients were diagnosed with sepsis. After applying exclusion criteria, 201 patients with sepsis were finally included in the sepsis group (Figure 1). Approximately, 213 patients with HF were included in the HF group, including 92 patients from the ICU and 121 patients from the cardiology department. Approximately, 211 healthy subjects were included in the control group. A total of 625 participants were included in the study. Table 1 shows the baseline demographic and disease characteristics of the participants.

**Table 1:** Baseline characteristics of participants.

**Characteristics**	**Sepsis group** **(*n =* 201)**	**Heart failure group** **(*n =* 213)**	**Control group** **(*n =* 211)**	***P/*** **χ^2^**
Gender (male), *n* (%)	112 (55.7)	119 (55.9)	116 (55.0)	0.854
Age, median (IQR), *y*	66 (57–75)	68 (57–78)	67 (57–73)	0.191
**Complication, *n* (%)**				
Hypertension		116 (54.4)		
CHD		126 (59.2)		
Valvulopathy		58 (27.2)		
Cardiomyopathy		18 (8.5)		
Diabetes	75 (37.3)	97 (45.5)		0.090
Cerebrovascular disease	29 (14.4)	20 (9.4)		0.117
CKD	30(15.0)	32 (15.0)		0.987
Tumor	9(4.4)	6 (2.8)		0.377
COPD	62 (30.8)	60 (28.1)		0.550
Alimentary tract hemorrhage	69 (34.3)	39 (15.0)		<0.001^**^
**Infection site, *n* (%)**				
Lower respiratory infection	110 (54.7)			
Abdominal infection	59 (29.4)			
Skin soft-tissue infection	5 (2.5)			
Urinary infection	8 (4.0)			
Bloodstream infection	11 (5.5)			

*IQR, indicates interquartile range; CHD, coronary heart disease; CKD, chronic kidney disease; COPD, chronic obstructive pulmonary disease;*

** *represents p < 0.001*.

As shown in Table 1, there was no statistically significant difference in age and sex composition of the participants in the control group, the sepsis group, and the HF group, and the three groups were comparable. There was no significant difference in comorbidities between the sepsis group and the HF group, except for gastrointestinal bleeding, which was significantly higher in patients with sepsis than in patients with heart failure at the time of enrollment.

Among 201 patients with sepsis, 116 patients had lower respiratory tract infections. Fifty-nine patients had an abdominal infection, including 11 patients with gastric and duodenal perforation, 21 patients with large intestine perforation, 6 patients with intestinal obstruction and intestinal necrosis, 3 patients with liver abscess, 2 patients with suppurative appendicitis, and 16 patients with severe pancreatitis. There were 5 patients with skin and soft tissue infection, including 2 patients with a wound infection after amputation caused by trauma, 2 patients with pressure ulcer infection, and 1 patient with facial cellulitis caused by periodontitis and mandibular arthritis. Eight patients had urinary tract infections. Eight patients had brain infections, and 19 patients had bloodstream infections.

### Differences in Cardiac Indicators Among Patients With Sepsis, Patients With Heart Failure, and Healthy Individuals

Bonferroni test was used to compare data among the three groups. As shown in Table 2, compared to healthy people, HR was significantly increased and LVEF was significantly decreased in patients with sepsis and HF (*p* < 0.001). However, there was no significant difference in HR and LVEF between the sepsis and HF groups, *p* (HR) = 0.179, *p* (LVEF) = 0.238.

**Table 2:** Multiple comparisons of cardiac indicators among the sepsis group, the HF group, and the control group.

**Variables**	**Control group** **(*n =* 211)**	**Sepsis group** **(*n =* 201)**	**HF group** **(*n =* 213)**	***p***
HR (beat/minute)	71.4 ± 9.4	85.3 ± 11.9^*^	88.1 ± 14.1^*^	0.000
LVEF (%)	61.7 ± 9.4	44.3 ± 8.7^*^	42.8 ± 8.3^*^	0.000
BNP(pg/ml),median	NA	480.0	570.0	0.046
(IQR)		(310.0, 690.0)	(367.0, 772.5)^#^	
Tp-Te interval (ms)	91.3 ± 12.5	115.3 ± 23.3^*^	118.9 ± 19.9^*^	0.000
Tp-Te/QT	0.216 ± 0.057	0.291 ± 0.058^*^	0.330 ± 0.05183^*#^	0.000

*Data are expressed as mean ± SD or Median (25%, 75%); IQR, indicates interquartile range; HF, heart failure; HR, heart rate; LVEF, left ventricular ejection fraction; BNP, brain natriuretic peptide; P, the statistical P-value for comparison among the three groups*.

As compared with the control group, ^*^ represents p < 0.05;

*As compared with the sepsis group, ^#^ represents p < 0.05*.

The Tp-Te interval and Tp-Te/QT in patients with sepsis and HF were significantly higher than in the control group (*p* < 0.001). Compared with patients with sepsis, the increase of Tp-Te interval and Tp-Te/QT ratio is more prominent in patients with HF. However, the difference of Tp-Te interval between the two groups was not statistically significant (*p* = 0.143). The Tp-Te/QT ratio difference was statistically significant (*p* < 0.001).

Patients with sepsis have less BNP value than patients with HF, and showed statistical significance (*p* < 0.05).

### The Differences Between Sepsis Patients With Different Levels of Severity

We divided patients with sepsis into subgroups: 72 patients with septic shock were included in the septic shock group; moreover, 129 patients without shock were included in the sepsis (no shock) group (Table 3).

**Table 3:** Comparison between the sepsis (no shock) group and the septic shock group.

**Variables**	**Sepsis (no shock)** **group (*n =* 129)**	**Septic shock group** **(*n =* 72)**	***p***
Tp-Te interval (ms)	110.2 ± 22.2	124.4 ± 22.3	<0.001^**^
Tp-Te/QT	0.255 ± 0.030	0.356 ± 0.020	<0.001^**^
LVEF(%)	45.1 ± 9.3	43.0 ± 7.3	0.085
BNP(pg/ml), median (IQR)	496.0(356.0, 690.0)	549.0(300.0, 856.0)	0.880
SOFA	7.1 ± 2.9	11.7 ± 2.1	<0.001^**^
APACHE-II, median (IQR)	17.0(13.0, 23.5)	19.0(13.0, 25.0)	0.089

*Data are expressed as mean ± SD or Median (25%, 75%); IQR indicates interquartile range; LVEF, left ventricular ejection fraction; BNP, brain natriuretic peptide; SOFA, organ failure assessment; Apache-II, acute physiology and chronic health evaluation;*

** *represents p < 0.001*.

According to Table 3, compared with patients with sepsis (no shock), the Tp-Te interval, Tp-Te/QT ratio, and SOFA were increased in patients with septic shock (*p* < 0.05). There was no significant difference in LVEF, BNP, and Apache-II scores between the two groups.

### The Difference Between Patients With Sepsis With Different Outcomes

We divided patients with sepsis into subgroups based on outcomes. Death within 28 days was considered the outcome event. The patients who died within 28 days were included in the death group. The patients who improved or were discharged from ICU within 28 days were included in the survival group. Among 201 patients with sepsis, 22 patients gave up treatment and signed out of the hospital. Approximately, 179 patients were included in this subgroup study; 51 died and 128 survived (Table 4).

**Table 4:** Comparison between the survival group and the death group.

**Variables**	**Survival group** **(*n =* 128)**	**Death group** **(*n =* 51)**	***p***
Tp-Te interval (ms)	113.3 ± 23.2	116.4 ± 21.5	0.421
Tp-Te/QT	0.271 ± 0.05	0.336 ± 0.06	<0.001^**^
LVEF (%)	45.09 ± 8.83	41.42 ± 7.11	0.009^*^
BNP(pg/ml), median (IQR)	490.0 (305.00, 690.0)	580.0 (430.5, 692.0)	0.140
SOFA	7.9 ± 3.3	10.5 ± 3.1	<0.001^**^
APACHE-II, median (IQR)	17.0(13.0, 24.0)	18.0(14.0, 25.0)	0.039^*^

*Data are expressed as mean ± SD or Median (25%, 75%); IQR indicates interquartile range; LVEF, left ventricular ejection fraction; BNP, brain natriuretic peptide; SOFA, organ failure assessment; Apache-II, acute physiology and chronic health evaluation; data are expressed as mean ± SD;*

** *represents p < 0.001*.

* *represents p < 0.05*.

Table 4 shows that the BNP, Tp-Te, Tp-Te /QT, SOFA, and Apache-II of the death group were higher than the survivors, while the LVEF of the death group was lower than the survivors. The differences of LVEF, Tp-Te/QT, SOFA, and Apache-II between the two groups were statistically significant (*p* < 0.05).

### Predictive Value of Tp-Te Interval and Tp-Te/QT Ratio in Patients With Sepsis

**Figure 2:**
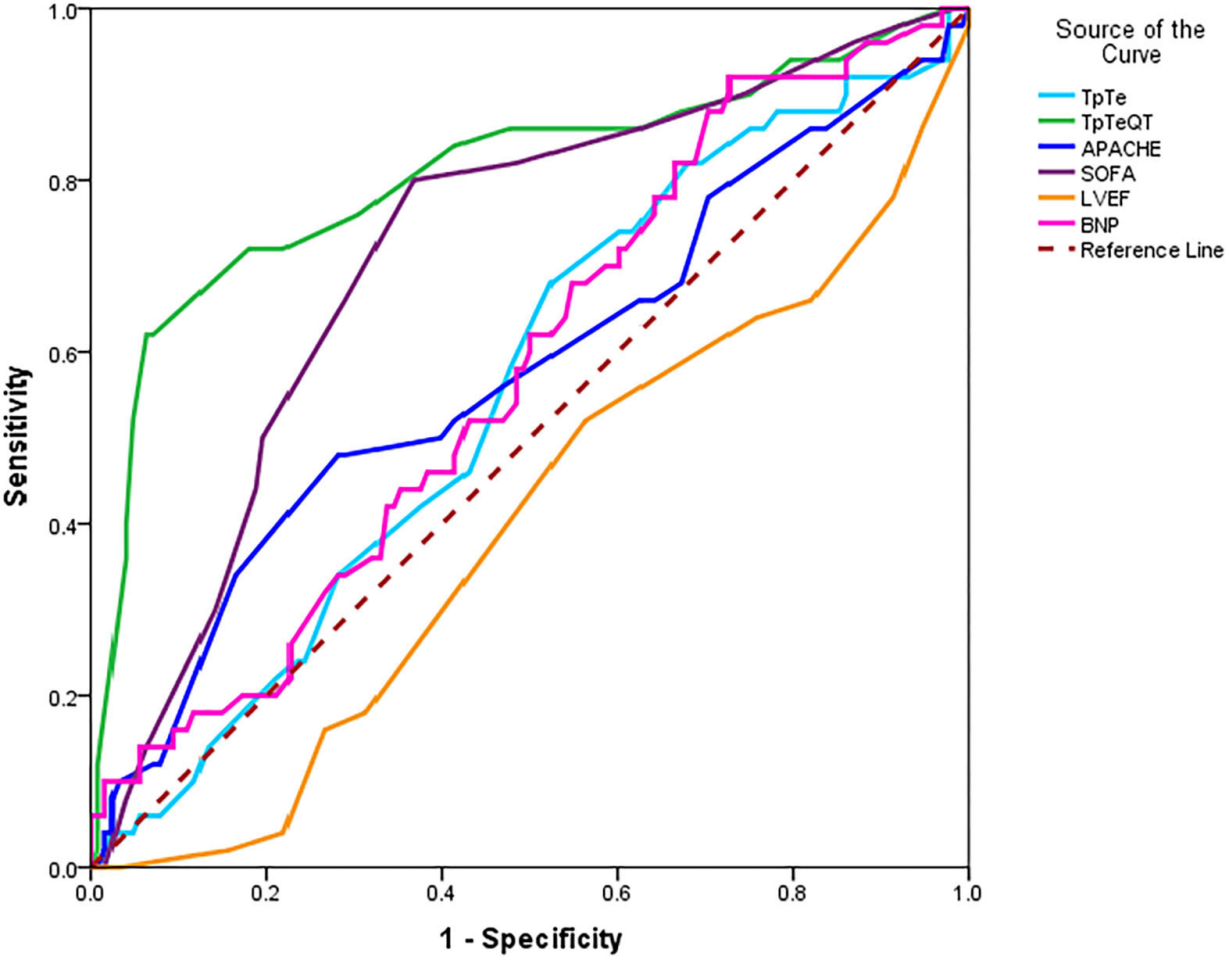
ROC curves of different indicators in sepsis patients.

We used ROC curves to analyze the predictive value of clinical indicators in the patients with sepsis and explored whether the electrocardiology index has advantages over the traditional indicators (Table 5 and Figure 2).

**Table 5:** Analysis of the predictive value of clinical indicators by ROC curve.

**Variables**	**AUC**	***p***
Tp-Te interval (ms)	0.556	0.243
Tp-Te/QT	0.808	<0.001^**^
LVEF (%)	0.412	0.04^*^
BNP (pg/ml)	0.578	0.105
SOFA	0.716	<0.001^**^
APACHE-II	0.599	0.102

*AUC, area under curve; LVEF, left ventricular ejection fraction; BNP, brain natriuretic peptide; SOFA, organ failure assessment; Apache-II, acute physiology and chronic health evaluation*.

** *represents p < 0.001*.

As shown in Table 5, Figure 2, in the above six forecast indicators, Tp-Te/QT, SOFA, and LVEF have predictive death values (*p* < 0.05). However, only the AUC of Tp-Te/QT and SOFA were higher than 0.7, indicating that both of them have good prediction accuracy. Moreover, the AUC of Tp-Te/QT is closer to 1, with the optimal predictive value (Note: The AUC of LVEF is <0.5 because it is contrary to other indicators, and lower LVEF has a poorer prognosis.).

### The Cut-Off Point of Tp-Te/QT

Using ROC curve results, calculate the maximum value of (specificity + sensitivity); the corresponding Tp-te/QT is the cutoff point value of Tp-Te/QT. Thus, the cutoff point of Tp-Te/QT is 0.32. This means that the optimal Tp-Te/QT cutoff for predicting death in patients with sepsis is 0.32.

### Correlation Analysis of Tp-Te/QT With SOFA and LVEF

**Figure 3:**
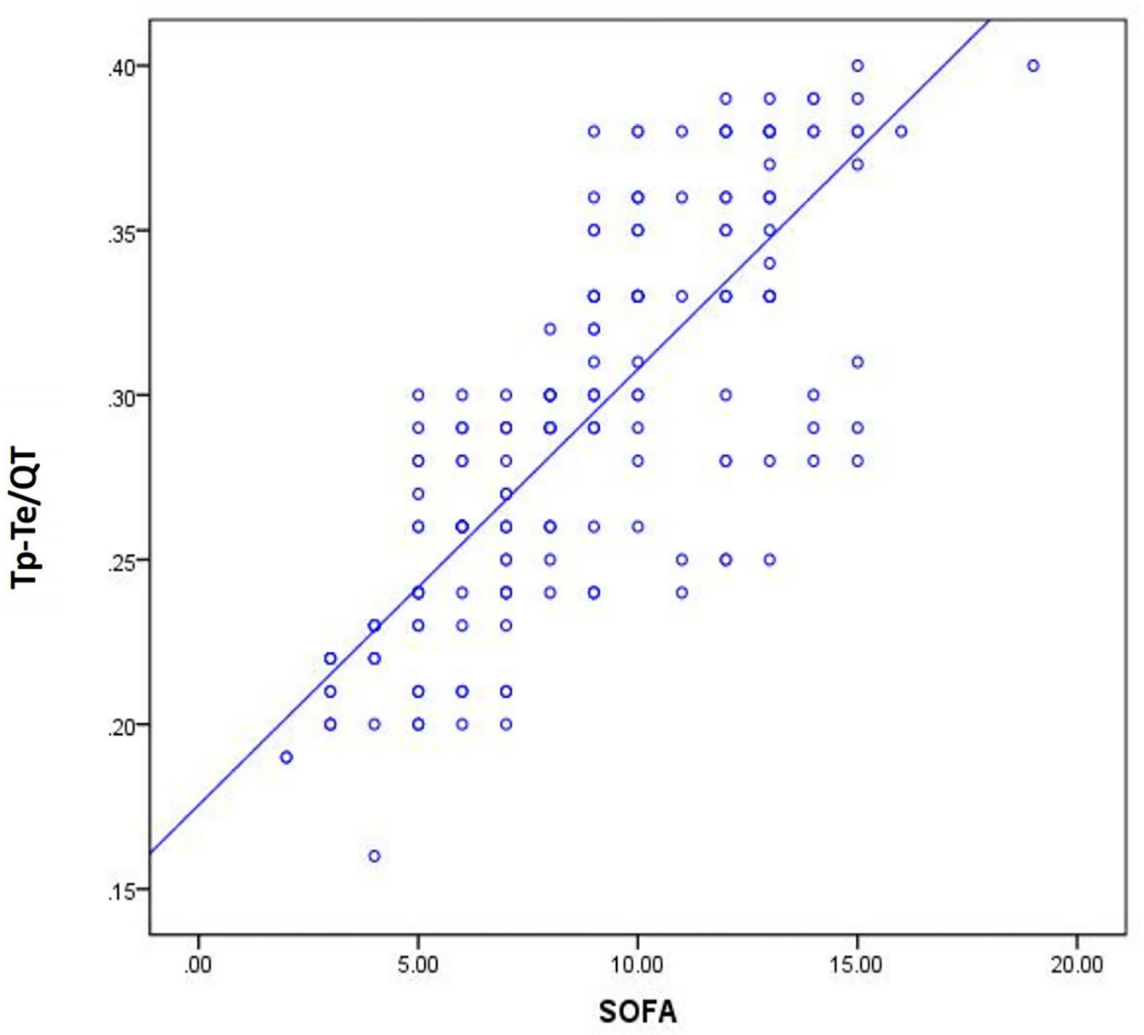
Scatter plot of correlation between Tp-Te/QT and SOFA.

We found that Tp-Te/QT, SOFA, and LVEF have predictive value for the death of patients with sepsis; thus, we discuss the correlation between the electrocardiology-index Tp-Te/QT and the traditional clinical indicators SOFA and LVEF (Table 6 and Figure 3).

**Table 6:** Correlation analysis of Tp-Te/QT with SOFA and LVEF.

**Variables**	**r**	***p***
Tp-Te/QT and SOFA	0.79	<0.001^**^
Tp-Te/QT and LVEF	−0.09	0.211

*SOFA, organ failure assessment; LVEF, left ventricular ejection fraction; r, correlation coefficient*.

** *represents p < 0.001*.

Table 6, Figure 3 show a significant correlation between Tp-Te/QT and SOFA, but no correlation was found between Tp-Te/QT and LVEF.

### Incidence of Arrhythmia in Patients With Sepsis With Different TDRs

According to the cutoff value of Tp-Te/QT, patients with sepsis are divided into subgroups. Among 201 patients with sepsis, 140 patients had Tp-Te/QT < 0.32, and 61 patients had Tp-Te/QT ≥ 0.32. Table 7 shows the incidence of arrhythmia in patients with sepsis with different TDRs.

**Table 7:** Incidence of arrhythmia in patients with sepsis with different TDRs.

**Groups**	**patients with newarrhythmia *n* (%)**	**Patients without arrhythmia** ***n*** **(%)**	**Total**
Tp-Te /QT < 0.32	23 (16.4%)	117 (83.6%)	140
Tp-Te /QT ≥ 0.32	27 (44.3%)	34 (55.7)	61
Total	50	151	201

*Results of Pearson Chi-Square test: χ^2^ = 17.61, p < 0.001*.

Among 201 patients with sepsis, 50 patients (24.9%) had new arrhythmia. According to Table 7, the incidence of new arrhythmia in the patients with sepsis with smaller TDR (Tp-Te/QT < 0.32) was significantly lower than the patients with larger TDR (Tp-Te/QT ≥ 0.32), and there was a statistical difference in the incidence of arrhythmia between the two groups.

### Types of New Arrhythmia in Patients With Sepsis With Different TDRs

New arrhythmias were defined as the arrhythmia that had not occurred prior and first occurred after admission to ICU, excluding sinus tachycardia, incidental atrial and ventricular premature beats (these arrhythmias can occur under normal physiological conditions). Electrocardiology data were collected prior to drug therapy. For the patients with persistent arrhythmias at the time of admission to ICU, we were not sure if the arrhythmias were preexisting, so we did not include them in the study.

**Figure 4:**
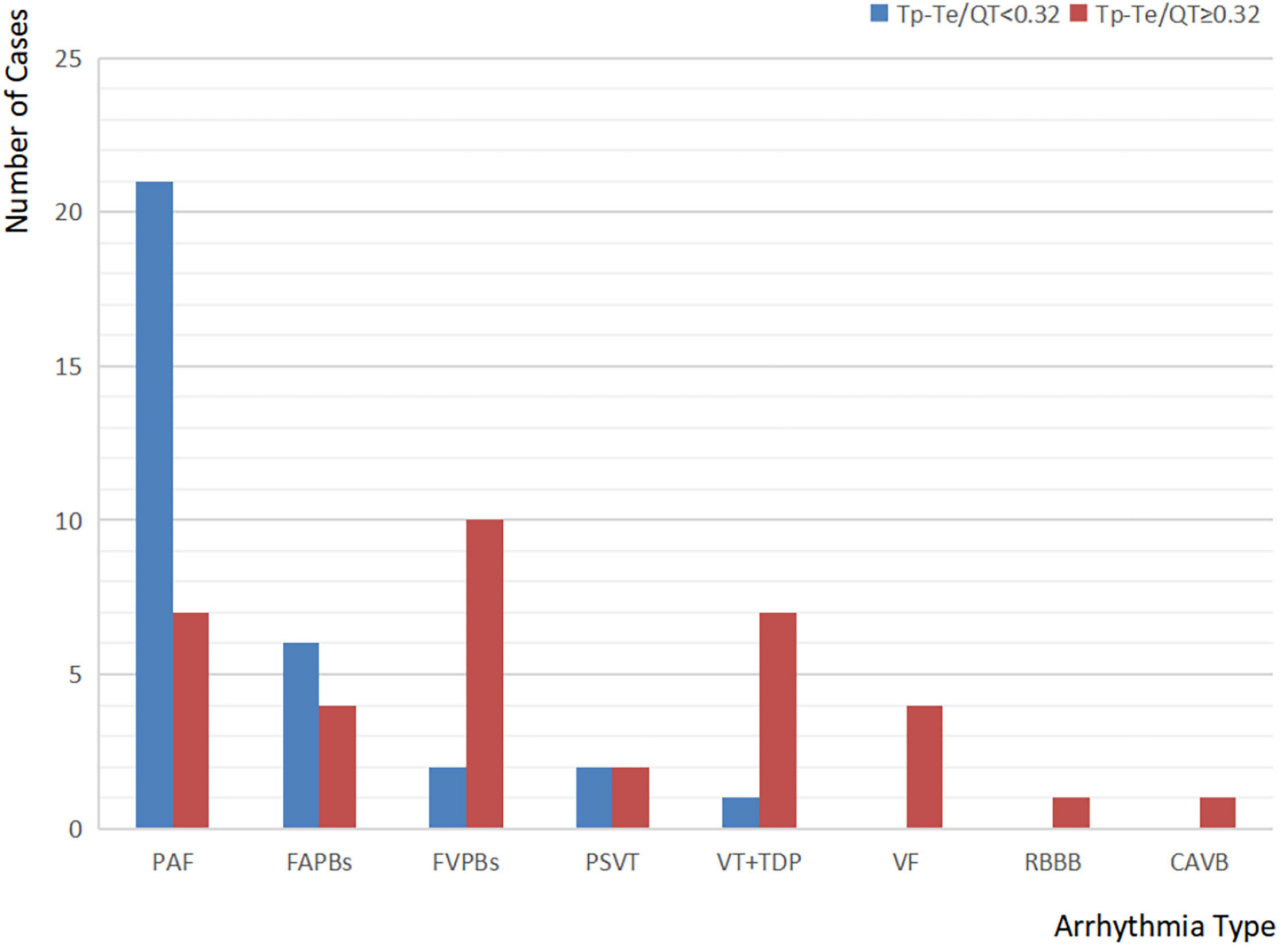
Types of new arrhythmia in patients with sepsis with different TDRs.

According to Table 8, Figure 4, the most common type of new arrhythmia among all the patients with sepsis was paroxysmal atrial fibrillation (PAF), which occurred in 28 cases, accounting for 56% of the patients with new arrhythmia. However, the distribution of arrhythmia categories is different in patients with sepsis with different TDRs. In patients with sepsis with Tp-Te/QT <0.32, the incidence of PAF was the highest, followed by frequent atrial premature beats (FAPBs). In patients with sepsis with Tp-Te/QT ≥ 0.32, the most common types of new arrhythmia were frequent ventricular premature beats (FVPBs), followed by ventricular tachycardia (VT), including torsade de pointes (TDP), and PAF. However, almost all arrhythmias that may affect hemodynamics, such as VT, TDP, ventricular fibrillation (VF), occurred in the patients with sepsis with Tp-Te/QT ≥ 0.32 (Note: The total number of arrhythmia cases is greater than the number of patients with arrhythmia, because some patients have two or more arrhythmias.).

**Table 8:** Types of new arrhythmia in patients with sepsis with different TDRs.

**Arrhythmia type**	**Sepsis patients with Tp-Te/QT <0.32 (*n =* 140)**	**Sepsis patients with Tp-Te/QT ≥0.32** **(*n =* 61)**	***p***
PAF, *n*	21	7	0.507
FAPBs, *n*	6	4	0.495
FVPBs, *n*	2	10	<0.001^**^
PSVT, *n*	2	2	0.586
(VT+TDP), *n*	1 + 0	5 + 2	0.001^*^
VF, *n*	0	4	0.008^*^
RBBB, *n*	0	1	0.303
CAVB, *n*	0	1	0.303

*PAF, paroxysmal atrial fibrillation; FAPBs, frequent atrial premature beats; PSVT, paroxysmal supraventricular tachycardia; FVPBs, frequent ventricular premature beats; VF, ventricular fibrillation; VT, ventricular tachycardia; TDP, torsade de pointes; RBBB, right bundle branch block; CAVB, complete atrioventricular block*.

*If all expected counts ≥5 and total sample size N ≥ 40, Pearson chi-square test was performed*.

*If there is an expected count <5, Fisher's Exact Test is used*.

** *represents p < 0.001*.

* *represents p < 0.05*.

## Discussion

Ventricular repolarization is a complex process. Since the 1990s, the electrophysiology of action potential duration (APD) in myocardial triply cells has been extensively studied, and the heterogeneous repolarization time of transmural cardiomyocytes was gradually revealed (5). Tp represents the end of epicardial repolarization, and Te represents the end of M cell repolarization. Tp-Te interval represents the transmural dispersion repolarization (TDR) (6). Abnormally enlarged TDR is the cause of many malignant ventricular arrhythmias and sudden cardiac death and an essential indicator for predicting the prognosis of patients with heart disease (17–19). Previous studies also confirmed that Tp-Te and Tp-Te/QT were increased in patients with HF (19, 20), and can be used as effective indicators to predict sudden cardiac death (SCD) in patients with HF.

Herein, we found that the TDR of patients with sepsis was significantly increased. This may indicate an increased risk of malignant arrhythmias and sudden cardiac death in patients with sepsis. However, the increase of TDR in patients with HF was more pronounced. The TDR of patients with sepsis is between a healthy person and patients with heart failure. We realized that the patients with HF in this study were all from the inpatient department, and the criterion for admission is that the patient's cardiac function is at Grades III–IV (NYHA). Probably, because the patients were sicker led to this result, if the proportion of Grades I–II patients were increased, the results may be different.

In ICU, Apache-II and SOFA are the most widely used indexes to evaluate the prognosis and severity of patients. BNP and LVEF are commonly used to reflect cardiac function. Comparing subgroups, we explored the differences among patients with sepsis with different severity and different prognoses. We found that the patients with septic shock had higher Tp-Te, Tp-Te/QT, and SOFA scores. This result is not completely consistent with the findings in children with sepsis by Turkish Scholar Ozdemir R (13). The Ozdemir R study also confirmed that Tp-Te and Tp-Te/QT were significantly higher in the sepsis group than in the control group. There were no significant differences in Tp-Te interval and Tp-Te/QT between the sepsis group, the severe sepsis group, and the septic shock group in the Ozdemir R study. We also found that the Tp-Te/QT and SOFA in patients with sepsis who died within 28 days were significantly higher, and the LVEF was lower than those who survived. This indicates that patients with sepsis with different degrees of severity have different transmural dispersion repolarization of the heart. Patients with more severe disease and poorer prognosis have a higher TDR.

Our research found that there was no correlation between Tp-Te/QT and LVEF, possibly because the clinical manifestations of SMD are very complex, including systolic failure and diastolic failure. It can be either left ventricular and right ventricular failure or in combination. LVEF simply reflected the ventricular systole function. However, the lengthening of Tp-Te/QT reflected the changes in the overall heart. Therefore, there is no significant correlation between them.

Our results also confirmed that the patients with greater TDR had a significantly increased rate of arrhythmia. Although PAF is the most common arrhythmia in the patients with sepsis, there was no statistical difference in its incidence between the Tp-Te/QT < 0.32 group and the Tp-Te/QT ≥ p-T2 group; the incidences of FVPBs, VT (including TDP), and VF were significantly different between the two groups. And almost all arrhythmias affecting hemodynamics occurred in patients with sepsis with Tp-Te/QT ≥ 0.32. These results suggest that the increase of TDR is an important cause of malignant ventricular arrhythmias. The greater TDR increases the risk of sudden cardiac death (SCD). This may be the reason why Tp-Te/QT has a good predictive value of death in patients with sepsis. Yayla et al. (21) studied 151 patients with frequent PVCs, and found that, before radiofrequency ablation, there was a significant correlation between Tp-Te/QTc ratio and PVC burden (*p* = 0.023). After the successful procedure, Tp-e interval, Tp-e/QT ratio, and Tp-e/QTc ratio significantly decreased (all *p* < 0.001). These results suggest that TDR increased in patients with high VPC number, VPCs could have a negative effect on myocardial repolarization, and the interaction could lead to an increased risk of malignant arrhythmias. This is consistent with the findings of our study.

However, the prediction effect of Tp-Te/QT on death is better than SOFA, which let us think that whether this conclusion is reliable? This study collected SOFA and Apache-II from the worst results within 48 h of admission. However, the score changes as patients progress with treatment. Scores at different time points may have different predictive values for poor outcomes. For example, the predictive value of a patient's SOFA score before death may be better than that of 48 h at admission. Therefore, in the clinical setting, we should still observe and rigorously evaluate the results vigorously to predict the poor prognosis of patients.

In addition, there was only a primary outcome (28-day all-cause death) in the study. If a secondary outcome is added, maybe, the results will be different. Furthermore, given the particularity of ICU patients, all cardiac ultrasound needs to be done at the bedside in ICU. Due to portable bedside cardiac ultrasound limitations, cardiac ultrasound indicators in many patients with sepsis are not comprehensively reported. If added to the study of cardiac ultrasound indicators, such as *E*/*E*′, LVIDD, it may be of great significance to explore the mechanism of TDR increase in patients with sepsis. Therefore, further studies are needed to confirm them.

## Conclusion

TDR in patients with sepsis is significantly increased between healthy population and patients with HF. Tp-Te interval and Tp-Te/QT, as the electrocardiogram- indicators of TDR, reflected the severity and a poor outcome of patients with sepsis. Patients with sepsis with the greater TDR had an increased incidence of malignant arrhythmia. In addition, Tp-Te/QT has a significant correlation with SOFA.

## Data Availability Statement

The raw data supporting the conclusions of this article will be made available by the authors, without undue reservation.

## Ethics Statement

The protocol used in this study was approved by the Medical Ethics Committee, Beijing Fuxing Hospital, Capital Medical University (2013FXHEC-KY018). The patients/participants provided their written informed consent to participate in this study.

## Author Contributions

DL was involved in the acquisition of data, data processing, study design, statistical analysis, and manuscript writing and drafting. YW was involved in the study design and data processing. GZ was involved in acquisition of data. LJ were involved in the study design and the final revision of the manuscript. All authors read and approved the final manuscript.

## Funding

This work was supported by the National Science and Technology Support Program (2012BAI11B05).

## Conflict of Interest

The authors declare that the research was conducted in the absence of any commercial or financial relationships that could be construed as a potential conflict of interest.

## Publisher's Note

All claims expressed in this article are solely those of the authors and do not necessarily represent those of their affiliated organizations, or those of the publisher, the editors and the reviewers. Any product that may be evaluated in this article, or claim that may be made by its manufacturer, is not guaranteed or endorsed by the publisher.
